# Development of a stability indicating high-performance liquid chromatography method for determination of cenobamate: study of basic degradation kinetics

**DOI:** 10.1186/s13065-024-01177-4

**Published:** 2024-04-13

**Authors:** Samah F. EL-Malla, Fotouh R. Mansour, Almoataz Bellah B. Elbastawissy, Samar H. Elagamy

**Affiliations:** Department of pharmaceutical analytical chemistry, Faculty of pharmacy, Tanta University, Tanta, Egypt

**Keywords:** Cenobamate, Impurity, Stress degradation, kinetics

## Abstract

This study presents a stability indicating high-performance liquid chromatography HPLC method for the determination of cenobamate (CNB) in presence of its main impurity (CNB H-impurity) and degradation products. The chromatographic separation was carried out on a Thermo BDS Hypersil-C18 column (150 × 4.6 mm; 5 μm) with a mobile phase consisting of a 50:50 (%*v/v*) ratio of methanol and purified water. The flow rate was maintained at 1.0 mL. min^− 1^. CNB was detected at 210 nm using a PDA detector. The column temperature was held at 40 °C.The retention time of the drug was found to be 3.2 min. Furthermore, the study investigates the degradation behavior of CNB under various stress conditions, including acidic, basic, oxidative, and light-induced degradation. The results indicate that CNB is particularly susceptible to basic degradation. Consequently, a comprehensive study of the basic degradation kinetics was conducted. The method was also successfully applied for the determination of CNB in its dosage form. The results also show that there is no co-elution from degradation products or excipients as indicated by the mass balance and peak purity values confirming the specificity of the proposed method and its applicability for routine analysis of CNB.

**Supplementary Information:**

The online version contains supplementary material available at 10.1186/s13065-024-01177-4.

## Introduction

Cenobamate, CNB, [(1R)-1-(2-chlorophenyl)-2-(tetrazol-2-yl)ethyl] carbamate is a novel antiepileptic drug developed by SK Life Science (SK) for treatment of partial onset seizures in adults and has been approved by FDA on November 2019 (Figure S1-a).The mechanism of action for CNB is still unclear, however some studies suggest that CNB can inhibit sodium channels resulting in reduction in repetitive neuronal firing. It is also a positive allosteric modulator of gamma-aminobutyric acid ion GABA channel [1–3]. The drug has Log P of 0.456 and it is slightly soluble in aqueous media over the pH range 1 to 8 [4]. CNB has an organic related impurity, CNB Hydroxy impurity (CNB H-impurity) which is an intermediate in the chemical synthesis of CNB (Figure S1-b) [5]. Few analytical methods has been reported for analysis of CNB including liquid chromatographic methods [6–8] and tandem mass spectrometric methods [9–11].

The study of forced degradation in pharmaceutical industry involves subjecting the drug to various stress conditions such as hydrolysis, oxidation, and photolysis. The specific stress levels applied should be customized based on the chemical characteristics of the active pharmaceutical ingredients (API), product type, and storage requirements, aiming for a degradation range of 5 to 20%, as exceeding this range would be unrealistic [12]. Forced degradation studies are important as they provide information about stability of pharmaceutical products in different environmental conditions such as relative humidity, light, and temperature and they help in identifying potential degradation pathways and impurity profiling [13, 14]. The study of degradation kinetics are also essential for establishing the optimal storage conditions and predicting the shelf life of drug substances under different environmental conditions. Kinetic parameters, such as rate constant, half-life (*t*_*1/2*_), time taken for 10% degradation (*t*_*90*_), and activation energy (*E*_*a*_), are utilized to determine the degradation order of the drug [15–19]. Degradation reactions of pharmaceuticals are typically classified as zero, first, or second order. Most pharmaceutical products undergo degradation via first-order or pseudo-first order reactions. Careful selection of variables for each degradation reaction is crucial as it provides insight into the rate-limiting factors. Typically, analyzing one variable at a time is important for better understanding of the individual variable’s impact on the kinetics of each degradation reaction [20, 21].

Among the reported methods for the chromatographic separation of CNB, there are two stability indicating methods [6, 7]. However, both of these methods detected CNB at 270 nm, which makes tracking of the degradation of CNB really challenging due to lack of reasonable absorbance at this particular wavelength. Thus, the objective of this research is to develop an optimized, green, and validated stability indicating HPLC method for accurate determination of CNB in presence of CNB H-impurity and stress degradation products. The chromatographic conditions were carefully optimized in the presence of a potential organic impurity (CNB H-impurity). The assessment of the greenness of the proposed method was also conducted using two green metrics including analytical ecoscale and green analytical procedure index GAPI. Moreover, it is worth noting that this research represents, for the first time, a kinetic study of the basic degradation of CNB that has not been previously published in the literature.

## Experimental

### Instrumentation

The separation was performed using HPLC Agilent 1260 Infinity Series (Agilent Technologies, Inc. USA) equipped with sampler (G1329B), quaternary pump (G1311C), PDA-Detector (G1315D), and column oven (G1316A). Signal monitoring and data processing were carried out using the ChemStation software. The mobile phase was degassed using Elmasonic; S 300 H, Germany. A magnetic stirrer: Stuart; US152, UK and a water bath: Memmert; WNB22, Germany were also utilized.

### Materials and reagents

CNB (99.79% purity) and CNB-H impurity were provided from Metrochem API Pvt Ltd, India. Xcopri® tablets were supplied from SK life science, Inc. Paramus, NJ07652. The tablets are labeled to contain 100.0 mg CNB. Both acetonitrile and methanol were HPLC grade and obtained from Scharlau, Spain. Diluent is prepared by mixing acetonitrile and purified water in the ratio of 40:60, % *v/v*. Excipients used in preparation of spiked placebo include microcrystalline cellulose (FMC, USA), colloidal silicon dioxide, (Evonik, Germany), lactose monohydrate (DFE Pharma, Germany), sodium starch glycolate (JRS Pharma, USA), magnesium stearate (Italmatch, Italy), and film coating agents: FD&C Blue# 2/indigo carmine aluminum lake, iron oxide red, iron oxide yellow (Karma, Egypt), polyethylene glycol 3350 (IMCD, Egypt), polyvinyl alcohol-part hydrolyzed (East Hony, China), Talc (Elgomohorya, Egypt), and titanium dioxide (Kronos, USA). Hydrogen peroxide (Merck, Germany, 30%), hydrochloric acid (Scharlau, Spain, 36.5–38%) and sodium hydroxide pellets (Scharlau, Spain, 98.5%) were utilized in the study of degradation conditions.

### Chromatographic conditions

The chromatographic separations were performed using a Thermo BDS Hypersil-C18 (150 × 4.6 mm; 5 μm) column. The mobile phase consisted of a degassed mixture of methanol and purified water in a ratio of (50:50, % *v/v*). Prior to analysis, the drug samples were filtered on nylon simple pure syringe filter, 0.45 μm, 25 mm, (Chromtech, UK). The column temperature was kept at 40 ^0^C. A 10 µL injection volume was used, and the drug was detected at a wavelength of 210 nm. The flow rate of the mobile phase was set at 1.0 mL.min^− 1^, and the total run time for each analysis was 5.0 min.

### Standard solutions and calibration curve

#### CNB standard solution

The drug stock standard solution was prepared by weighing 100 mg of CNB, transferring into 100-mL volumetric flask, and dissolving in 70 mL of the diluent (acetonitrile: purified water 40:60, %*v/v*) using sonication for 5.0 min. The solution was then cooled to room temperature and brought to the final volume with the diluent to obtain a final concentration of 1000 µg. mL^− 1^. A working standard solution (100 µg. mL^− 1^) was subsequently prepared by diluting 5.0 mL of the previously prepared stock solution to 50 mL with the mobile phase.

To construct the calibration curve, different aliquots of CNB stock standard solution (0.2–10.0 mL) solution were quantitatively transferred into a series of 50 mL volumetric flasks and completed to volume with the mobile phase to get solutions in the range of 4.0 to 200.0 µg. mL^− 1^. The average peak areas (mAU) of triplicate measurements of each concentration at 210 nm was plotted versus CNB concentration (µg. mL^− 1^).

#### Standard solution of the impurity

To prepare the impurity stock solution (CNB H-impurity) with a final concentration of 100.0 µg. mL^− 1^, 10.0 mg of the impurity was accurately weighed and transferred into a 100 mL volumetric flask. It was then dissolved using the diluent, and the volume was completed to 100 mL with the diluent. The standard solution of the impurity was separately injected into the HPLC system to determine its retention times. To create a sample solution containing 100.0 µg. mL^− 1^ spiked with a H-impurity in the concentration of 0.2 µg. mL^− 1^, 5 mL of the stock standard solution of CNB (1000 µg. mL^− 1^) was taken and spiked with 100 µL of the impurity stock solution (100 µg. mL^− 1^) in a 50 mL volumetric flask. The volume was then adjusted to 50 mL with the mobile phase.

### Forced degradation studies

CNB was exposed to different stress conditions including acidic, alkaline, oxidative, heat and light induced degradation. The drug concentration in all stress studies was 100 µg.mL^− 1^. For each stress condition, a minimum of two samples were prepared. Peak purity tests were conducted, and mass balances were calculated for each of these stressed samples.

The mass balance was calculated using the following formula

$$\begin{array}{l}{\rm{The}}\,{\rm{mass}}\,{\rm{balance}}\, = \,\\\frac{{\% assay\,of\,stressed\,sample + \% assay\,of\,degradants}}{{\% assay\,of\,unstressed\,sample}} * 100\end{array}$$

To calculate the percent degradation in each stress experiment, the following equation was applied: `

$${\rm{\% }}\,{\rm{degradation}}\,{\rm{ = }}\,\frac{{{\rm{(}}{{\rm{P}}_{{\rm{ST}}}}{\rm{ - }}{{\rm{P}}_{{\rm{DEG}}}}{\rm{)}}}}{{{{\rm{P}}_{{\rm{ST}}}}}} * {\rm{100}}$$

where:

P_ST_: represents the average peak area of CNB in standard solution without degradation.

P_DEG_: represents the average peak area of CNB in the stress degradation solution.

Typically, the target degradation percentages for the validation of stability studies are generally set within the range of 5–20% for the drug substance. The optimal conditions for degradation procedures, including reagent strength, exposure time, and temperature, were determined for each type of stress degradation as follow:

#### Acid degradation

Acid degradation was performed by adding 1.0 mL of 6.0 M HCl to 5.0 mL of CNB stock standard solution (1000 µg. mL^− 1^) in 50-mL volumetric flask. The resulting mixture was left for 20 h at 80 ^0^C, allowed to cool to room temperature, neutralized with 1.0 mL of 6.0 M NaOH, and the volume was adjusted to the mark with the mobile phase.

#### Basic degradation

Basic degradation was conducted by adding 2.0 mL of 0.005 M NaOH to 5.0 mL of CNB stock standard solution (1000 µg. mL^− 1^) in 50-mL volumetric flask. The resulting mixture was left for 5.0 min at 60 ^0^C, allowed to cool to room temperature, neutralized with 0.005 M HCl, and the volume was adjusted with mobile phase.

#### Oxidative degradation

Oxidative degradation was performed by adding 2.0 mL of hydrogen peroxide (30%*v/v*) to 5.0 mL of CNB stock standard solution (1000 µg. mL^− 1^) in 50-mL volumetric flask at 60 ^0^C. The resulting mixture was left for 4 h and the volume was adjusted with the mobile phase.

#### Photo degradation

Light degradation was carried out by exposing 5.0 mL of drug stock solution (1000.0 µg. mL^− 1^) in 50 mL volumetric flask to direct sun light for 6 h and to lab fluorescent lightening (1070 lx.h in the visible region (400–800 nm) for 6 h. Then the volume was completed with mobile phase.

#### Thermal degradation

This testing was conducted to assess the drug’s stability under elevated temperatures. The testing was performed by transferring 5.0 mL of drug stock solution (1000 µg. mL^− 1^) into 50 mL volumetric flask, immersing it in a water bath maintained at temperature of 80 ^0^C for 6 h. Then, the volume was completed with mobile phase.

### Application to dosage form

To assess the CNB content in Xcopri®100 mg tablets, ten tablets were ground into a fine powder, and this mixture was carefully homogenized with a spatula. A quantity of powder equivalent to one tablet was placed in a 100mL volumetric flask, dissolved in 70 mL of diluent, and sonicated for 10 min with occasional shaking. After that, the volume was adjusted to the mark with the same diluent, resulting in a stock sample solution containing 1000 µg. mL^− 1^of CNB. Subsequently, a 5.0 mL portion from the stock solution was diluted to a 50 mL volumetric flask and completed to the mark with the mobile phase (sample solution claimed to contain 100 µg. mL^− 1^of CNB). This process was repeated for six test samples prepared from the ground tablets. These samples were then filtered and analyzed using procedures mentioned under Sect. 2.3. The CNB concentration in the tablets was determined based on the previously constructed calibration curve.

## Results and discussion

### Optimization of the chromatographic conditions

**Fig. 1:**
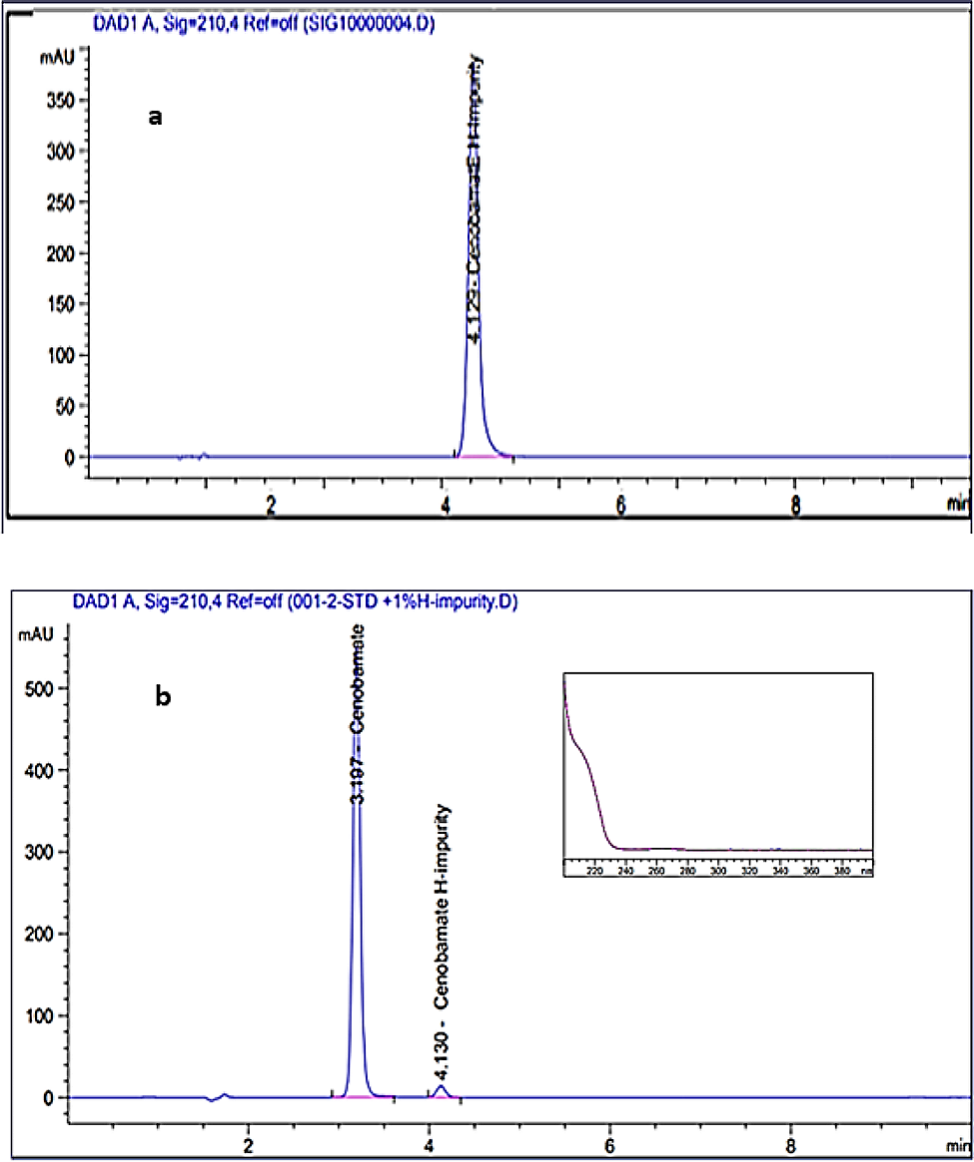
(**a**) The chromatogram of standard CNB H-impurity (100 µg.mL^− 1^) (**b**) The chromatogram of CNB (100 µg.mL^− 1^) spiked with CNB H-impurity (0.2 µg.mL^− 1^) and the extracted UV spectrum of the impurity

The solubility of CNB was evaluated in different solvents to identify the most suitable solvent for its analysis. Among the tested solvent combinations, (acetonitrile: purified water) 40:60%*v/v* shows better solubility than (methanol: purified water) 50:50%*v/v* respectively. Therefore, the diluent used for CNB analysis was acetonitrile to purified water (40:60% *v/v*). The HPLC method development and optimization was carried out based on the basis of system suitability parameters particularly the resolution between CNB, and its potential impurity CNB H-impurity. To optimize the chromatographic conditions, different stationary phases were studied including: Thermo Hypersil BDS C8 (150 × 4.6 mm, 5 μm), Thermo Hypersil BDS C18 (150 × 4.6 mm, 5 μm). The first column has a relatively lower hydrophobicity, and hence the latter gives a relatively high resolution between CNB, and CNB. H-impurity. Furthermore, various mobile phases containing different ratios of acetonitrile or methanol were tested. It was found that using methanol in mobile phase gives better resolution between CNB and CNB H impurity compared to acetonitrile. Additionally, the selected mobile phase (methanol: purified water, 50:50, %*v/v*) resulted in reasonable retention time, optimal resolution, number of theoretical plates, USP tailing factor, and capacity factor for CNB. This combination provided satisfactory results without necessitating the use of a buffer in the mobile phase, as the usage of buffer in different pH values in the mobile phase did not give any advanced chromatographic system suitability in comparison with purified water (Figure S2). The separation of CNB from its related organic impurity was also investigated. The developed chromatographic method demonstrated the capability of effective separation of CNB and its H-impurity. Table 1 shows the system suitability parameters for the optimum chromatographic condition for the separation of CNB and its impurity. The impurity was injected at a concentration of 100 µg. mL^− 1^ under optimized chromatographic conditions Fig. 1a. In accordance with USP guidelines, impurity levels should not exceed 0.2% of the active pharmaceutical ingredient (API) with total daily dose less than 2.0 g per day [22]. Figure 1b shows the chromatogram of CNB spiked with 0.2% of the impurity at optimum chromatographic conditions. Table S1 summarizes the system suitability parameters for all studied chromatographic conditions for separation of CNB and its organic related impurity.

**Table 1:** System suitability parameter for the optimum chromatographic condition for separation of CNB and its H-impurity

Condition	Retention time		Number of theoretical plates		USP Tailing factor		Resolution	Capacity factor (*k*) (CNB)
	CNB	H-impurity	CNB	H-impurity	CNB	H-impurity		
**Optimum condition**								
Thermo, Hypersil BDS C18 column (150 × 4.6 mm, 5 μm), mobile phase: Methanol: purified water 50:50, %*v/v*	3.2	4.14	7591	8369	1.06	1.04	5.8	2.56

### Stress degradation of CNB

The study was conducted to assess the stability and degradation mechanisms of CNB under various stress degradation conditions, which included acidic and basic hydrolysis, oxidative degradation, thermal stress, and photolysis. A summary of these stress conditions and the resulting percentage degradation is provided in Table S2.

**Fig. 2:**
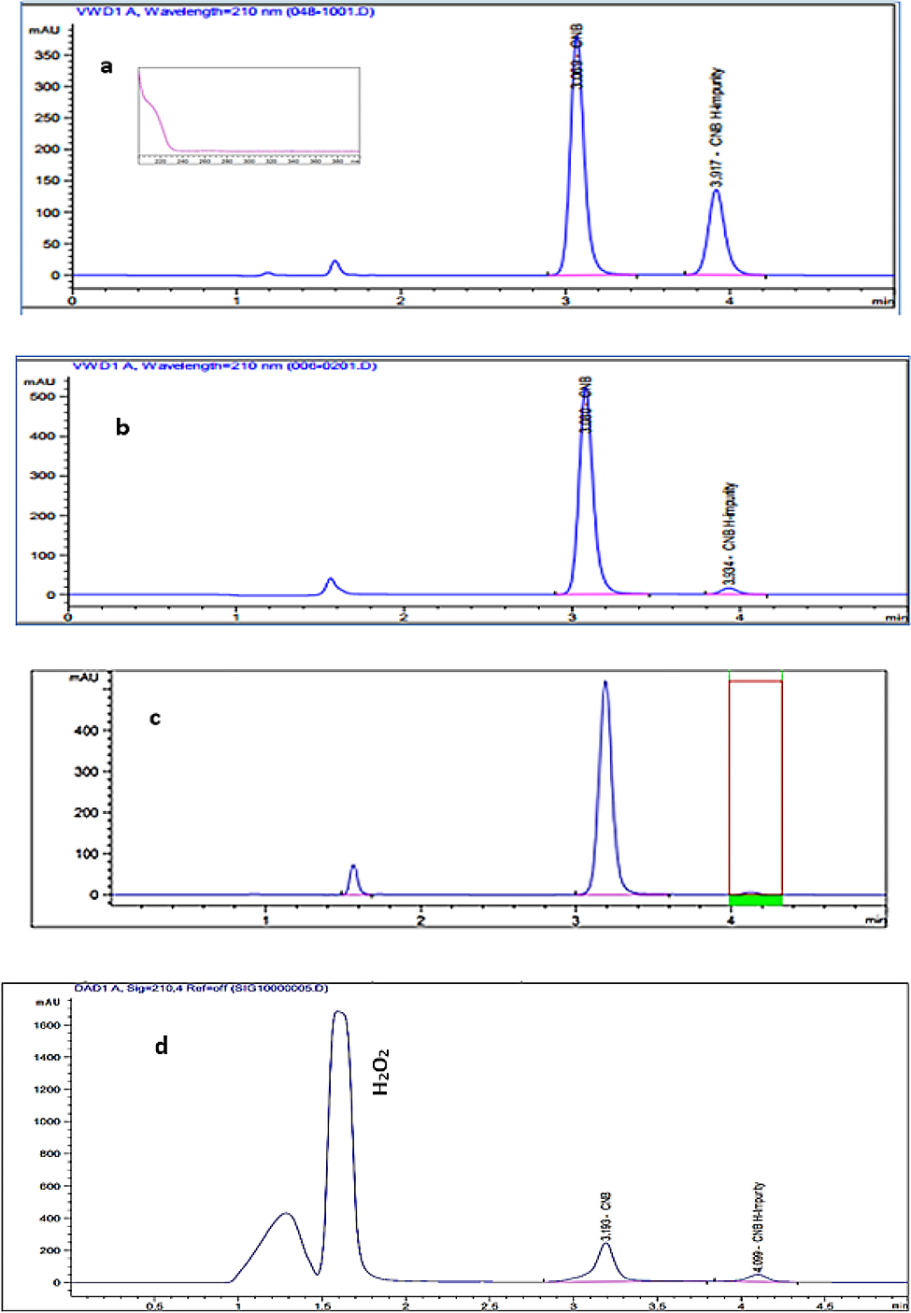
The chromatogram of CNB under (**a**) Alkaline stress using 0.005 M NaOH at 60^0^C for 5.0 min, (**b**) acidic stress using 6.0 M HCl at 80^0^C for 20 h, (**c**) thermal stress at 80^0^C for 6 h and (**d**) oxidative stress using 30% H_2_O_2_at 60^0^C for 4 h

**Fig. 3:**
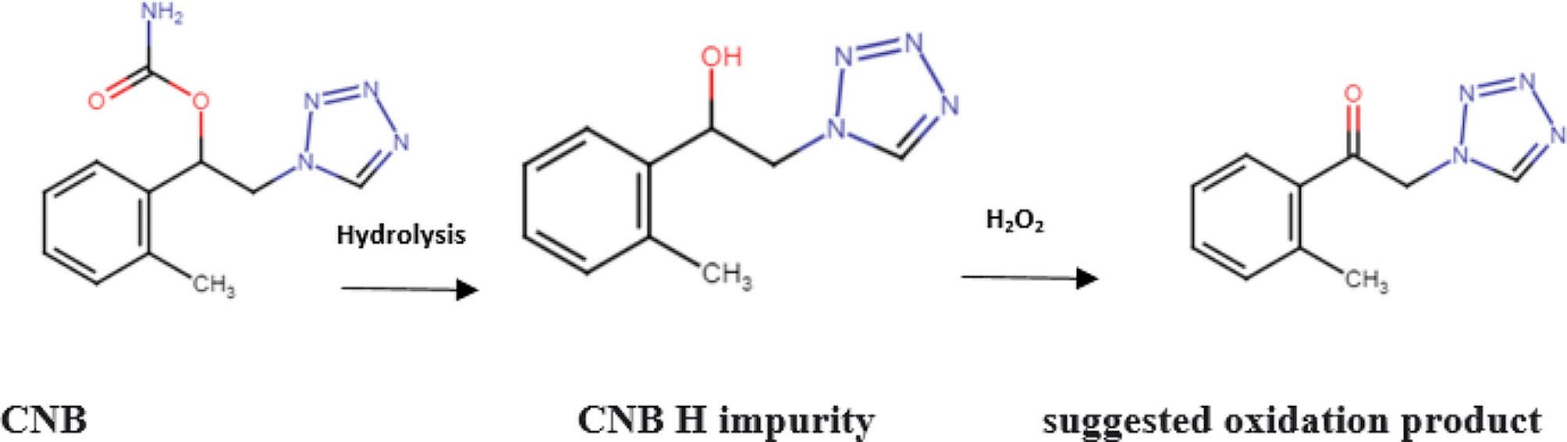
The proposed pathway for oxidative degradation of CNB

Significant basic degradation was achieved when heating CNB in 0.005 M NaOH for 5.0 min at 60 ^0^C. This could be attributed to the fact that CNB contains a carbamic acid esters (O-CO-NH-) which is susceptible to basic hydrolysis [23]. Chromatogram for basic hydrolysis of CNB show one degradation product peak which has the same retention time and extracted UV spectrum of the standard CNB H-impurity (Fig. 2a), confirming the identity of the alkali-induced degradation. In contrast, negligible acidic degradation (2.43% degradation) occurs at severe conditions using 6.0 M HCl after 20 h at elevated temperature 80 ^0^C yielding the same product for basic degradation suggesting that the acidic conditions could catalyze the breakdown of the carbamate moiety in CNB (Fig. 2b). Similarly, heat degradation leads to the formation of CNB H impurity (Fig. 2c). The oxidative degradation was observed under drastic conditions (30% H_2_O_2_, 4 h, 60^0^C) (Fig. 2d). This can be assigned to the formation of oxidative product of CNB-H impurity (Fig. 3). The suggested pathway is derived from the chemical structure of the drug and its impurity as the carbamate linkage in the drug is highly susceptible to cleavage under different stress condition as evidenced by the presence of CNB H impurity in the chromatogram which has hydroxyl group that can be easily oxidized to the ketone [23]. The formation of tetrazole N oxide was excluded as the tertrazole ring in the drug is substituted at position 1 which hinder its oxidation [24]. For photolytic stress, CNB did not undergo any degradation when exposed to direct sunlight or ordinary lab fluorescent lighting indicating its photostability.

The degradation study has been compared with a previously published stability indicating method [6], particularly regarding the % of oxidative and alkaline stress degradation Table S3. For all optimum stress conditions, the peak purity of CNB is greater than 0.999, and the mass balance results 95–99% (as shown in Table 2). This observation suggests the absence of interference or co-elution from degradants. Assay measurements for all the stressed samples were carried out in reference to a standard, enabling the calculation of mass balance.

**Table 2:** The percentage of degradation at the optimum degradation conditions with peak purity and mass balance assessment

Degradation conditions	Acid	Base	Heat	Oxidation
**Peak area (mAU)**	3237.831	2859.569	3003.310	3047.426
**Percentage remaining of CNB**	97.57	88.765	94.16	94.596
**Percentage of Degradation**	2.43	11.235	5.84	5.40
**Purity Factor**	999.907	999.920	999.869	999.877
**Mass balance (%)**	102	100.74	98.23	95

### Study of basic degradation kinetics

**Fig. 4:**
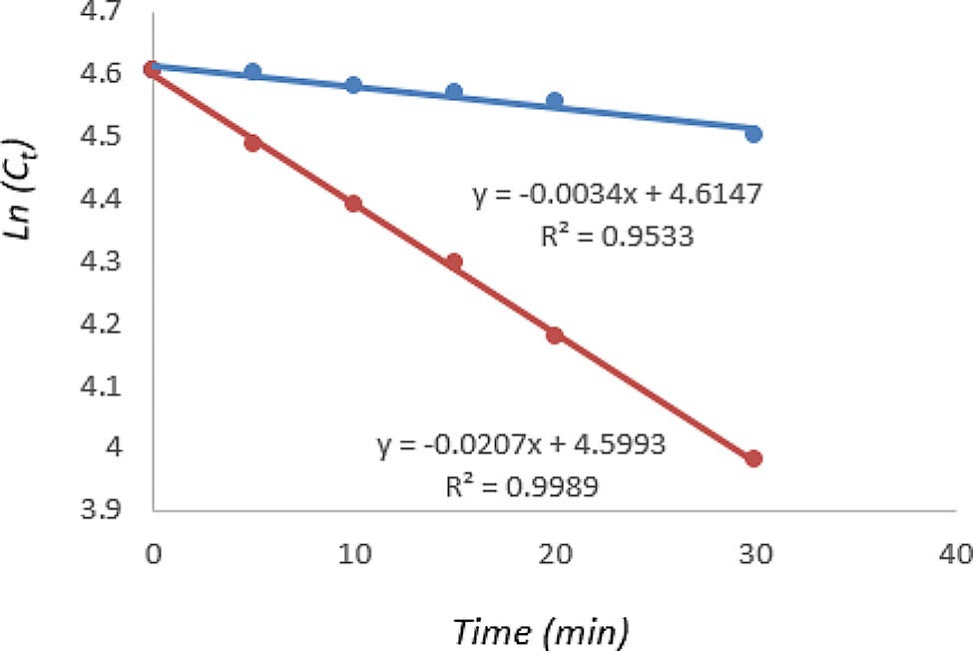
The kinetics plots of CNB basic degradation at different temperature (blue: 40 °C, red: 60 °C)

Since the stress degradation study revealed that CNB is highly susceptible to basic conditions, an investigation into the kinetics of basic degradation of CNB was conducted at 40 °C and 60 °C. The investigation involved the collection of samples at different time intervals (0, 5, 10, 15, 20, 30 min). Over time, a consistent decrease in the drug concentration was observed. At a higher temperature greater than 60 °C, the drug shows extensive degradation in the first five minutes. Notably, at temperatures of 40 °C and 60 °C, a linear graph was achieved when plotting *Ln(C*_*t*_) against time (Fig. 4) confirming that CNB follows a first-order kinetic model at these temperatures. The slope of the line is equal to the rate constant (*− K*).

The study also involved the calculations of degradation parameters, including the degradation rate constant (*K*), the time left for 50% potency (*t*_*1/2*_), and the time left for 90% potency (*t*_*90*_), according to the following equations:

$$ln\left[{C}_{t}\right]=-Kt+ln\left[{C}_{0}\right]$$

$${t}_{1/2}=\frac{0.693}{K}$$

$${t}_{90}=\frac{0.105}{K}$$

where *K* is the rate constant, [*C*_*0*_] is the concentration of CNB at time t = 0 and [*C*_*t*_] is the concentration at time (*t*).

The activation energy of CNB was determined using the Arrhenius equation, represented as follows:

$$Ln\frac{{K}_{1}}{{K}_{2}}=\frac{{E}_{a}}{R}\left(\frac{1}{{T}_{2}}-\frac{1}{{T}_{1}}\right)$$

In this equation, *K*_*1*_ and *K*_*2*_ are the basic degradation rate constants at 40 °C and 60 °C, respectively. *E*_*a*_ represents the activation energy, *R* is the gas constant (1.987 *cal mol*^*− 1*^*K*^*− 1*^, and *T*_*1*_ and *T*_*2*_ are the absolute temperatures in Kelvin *K* (313.15 *K*, 333.15 *K* respectively).

The calculated activation energy was found to be 18.72 *Kcal mol*^*− 1*^. With this value, it is possible to calculate the reaction rate at any other temperature [25]. The results of kinetic study are presented in Table 3.

**Table 3:** Results of the study of basic degradation kinetics of CNB

Temperature	40 ^0^C	60 ^0^C
**R** ^**2**^	0.9533	0.9989
**Slope**	-0.0034	-0.0207
***K *** **min** ^**-1**^	0.0034	0.0207
**t**_**½**_ **min**	203.823	33.478
**t**_**90**_ **min**	30.88	5.072

### Validation of the proposed method

The proposed HPLC method was validated according to ICH Q2 (R2), 2022 guidelines [26].

#### Linearity

The calibration graph exhibited linearity within the concentration range of 4.0 to 200.0 µg.mL^− 1^. Various statistical parameters, such as concentration ranges, linear regression equations, correlation coefficients, standard deviation of the intercept (S_a_), standard deviation of the slope (S_b_), and standard deviation of residuals (S_y/x_), were calculated and are presented in Table 4.

**Table 4:** The results of the regression parameters for determination of CNB by the proposed method

Parameters	
**Concentration range (µg. mL**^**− 1**^)	4-200
**Limit of detection (LOD) (µg. mL**^**− 1**^)	0.627
**Limit of quantitation (LOQ) (µg. mL**^**− 1**^)	1.901
**Regression Parameters** **Slope ± SD (S**_**b**_) **Intercept ± SD (S**_**a**_) **SD of residuals (S**_**y/x**_)	+ 31.119 ± 0.05969 -0.2681 ± 6.032467 1113.926
**Correlation coefficient (r)**	0.99997

#### Limit of quantitation and limit of detection

LOQ and LOD were calculated using the following formula:

$${\rm{LOQ}}\,{\rm{ = }}\,{\rm{10}}\,{\rm{\sigma /S}}$$

$${\rm{LOD}}\,{\rm{ = }}\,{\rm{3}}{\rm{.3}}\,{\rm{\sigma /S}}$$

In these equations, σ represents the standard deviation of the intercept, and S represents the slope of the calibration curve. For this study, the resulting LOD and LOQ were found to be 0.627 µg. mL^− 1^ and 1.901 µg. mL^− 1^, respectively Table 4.

#### Accuracy

The accuracy of the proposed method was evaluated by the determination of the mean percentage recovery, along with the standard deviation (SD), for three concentrations of CNB in pure form and spiked placebo. The results of this accuracy assessment are presented in Table S4.

#### Precision

In this study, repeatability (intra-day) was evaluated by conducting six replicate analysis of 100 µg. mL^− 1^ solution of CNB. Intermediate precision (inter-day) was determined by conducting repeated analysis of CNB using the chosen concentration over three successive days. The method’s precision was verified by observing low relative standard deviation (% RSD), as shown in Table S5.

#### Specificity and interference

The specificity of the proposed method was confirmed by Figure S3 which shows the chromatogram of CNB tablet, verifying that there is no interference from the tablet excipients. This was further confirmed by the peak purity spectra of CNB > 990 that are recorded using a PDA detector and by the high percentage recovery of the drug in tablet dosage form (99.14%) Table S6. The study also confirms the absence of interference from the primary impurity, CNB-H impurity, as it was introduced into the chromatographic system at concentrations of 0.2 µg. mL^− 1^ and distinctly separated at a retention time of 4.1 min, which is different from the retention time of CNB (3.2 min) (Fig. 1b).

#### Robustness

To evaluate the robustness of the procedure, minor alterations were introduced to the experimental parameters. These modifications encompassed a slight variation in the percentage of methanol within the mobile phase, ranging from 45 to 55%, as well as an adjustment in the flow rate by ± 5% (1.00 ± 0.05%). Despite these slight variations in the chromatographic conditions, the method remained unaffected, confirming its suitability for routine applications. The calculated pooled relative standard deviation (Pooled RSD %), using the formula Pooled RSD % = (average of SD/average of % Mean recovery) x 100, was found to be less than 3.0%, as presented in Table S7.

#### Stability of solution

The stability of the stock solution was assessed by comparing the responses of two standard solutions. The first solution was freshly prepared, whereas the second solution was diluted from a stock solution that had been prepared two days before and injected 48 h after its initial preparation. The findings indicate that the stock solution remains stable, permitting its use in sample preparation for a duration exceeding 24 h, as demonstrated in Table S8.

## Greenness assessment

**Fig. 5:**
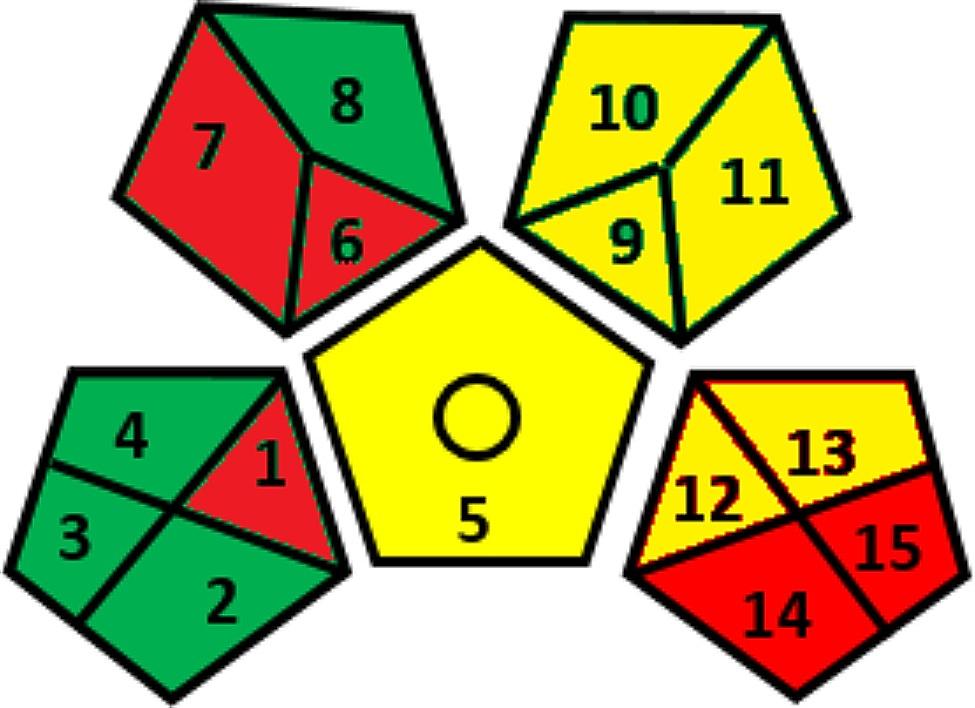
GAPI results for the developed HPLC method

The greenness of the developed method was evaluated using two common greenness metrics. The first metric is analytical ecoscale which is based on penalty points PPs assigned to different factors included in the analytical procedures, which are then subtracted from a base of 100. The score indicates the greenness of the analysis: more than 75 for excellent green analysis, more than 50 for acceptable green analysis, and less than 50 for inadequate green analysis. Factors such as reagent type and amount, the energy consumption of analytical equipment, the amount of the waste, the applicability of waste treatment, and occupational hazards are all considered and given penalty points [27]. The proposed method was found to be an acceptable green analytical method Table 5. The second metric is green analytical procedure index GAPI which is one of the most recent tools used to evaluate the greenness of analytical methods. It assesses the environmental impact of the entire method, from sample collection to final determination. This evaluation involves five pentagrams with a three-level color scale for each stage: green, yellow, or red, indicating high, medium, or low environmental impact, respectively [28]. Figure 5 shows the results of GAPI for the developed method. Table S9 provides explanation of GAPI results. It should be noted that the method could have been greener if a green solvent was used instead of methanol as a mobile phase modifier such as ethanol, acetone and glycerol [29–31].

**Table 5:** Greenness assessment of the developed method using analytical eco scale

**The developed HPLC method**	
**Reagents**	**PPs**
Purified water Methanol Acetonitrile	0 12 8 Ʃ20
**Instruments**	**PPs**
HPLC (≤ 1.5 kWh per sample) Occupational hazards Waste -Amount (> 10mL) - No treatment	1 0 5 3 Ʃ9
**Total PPs 29, score 71**	

## Conclusions

In conclusion, this study introduces a simple stability indicating HPLC method for the determination of CNB, along with an investigation of CNB basic degradation kinetics. By optimizing the HPLC conditions, CNB can be efficiently separated from its primary impurity and degradation by-products. Notably, the investigation revealed that CNB is extensively degraded under basic conditions. The results of the kinetic study of basic degradation indicated first-order kinetics. The method was also carefully validated regarding linearity, accuracy, precision, robustness, and specificity. Additionally, the developed method enables accurate CNB determination in tablet dosage form without any interference from excipients. The method also involves the utilization of purified water as a green and cost-effective solvent without the need for buffer solutions. Ultimately, this developed method has proven to be both specific and reliable, establishing its suitability for routine use in quality control laboratories for CNB analysis.

### Electronic supplementary material

Below is the link to the electronic supplementary material.

Supplementary Material 1

## Data Availability

All data generated or analysed during this study are included in this published article [and its supplementary information files].
