# Supplementary material for: Development of a stability indicating high-performance liquid chromatography method for determination of cenobamate: study of basic degradation kinetics
Source: BMC Chem. 2024 Apr 13;18(1):74. doi: 10.1186/s13065-024-01177-4 (PMC11016219; doi:10.1186/s13065-024-01177-4)
Supplement: Supplementary file 1 — Supplementary Material 1 [file 13065_2024_1177_MOESM1_ESM.docx]

**Supplementary information**

**Development of a stability indicating high-performance liquid chromatography method for determination of cenobamate:** **Study of basic degradation kinetics**


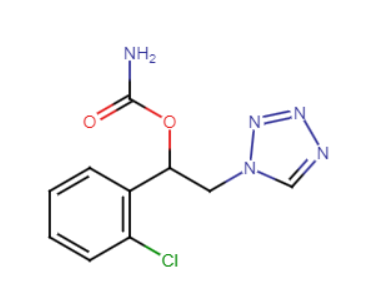

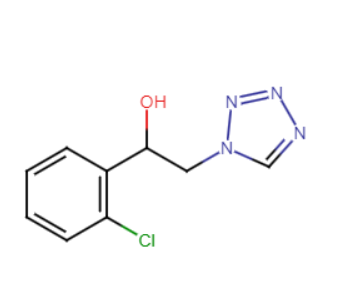


**(a) (b)**

## Figure S1: Chemical structure of CNB (a), and CNB H-impurity (b).


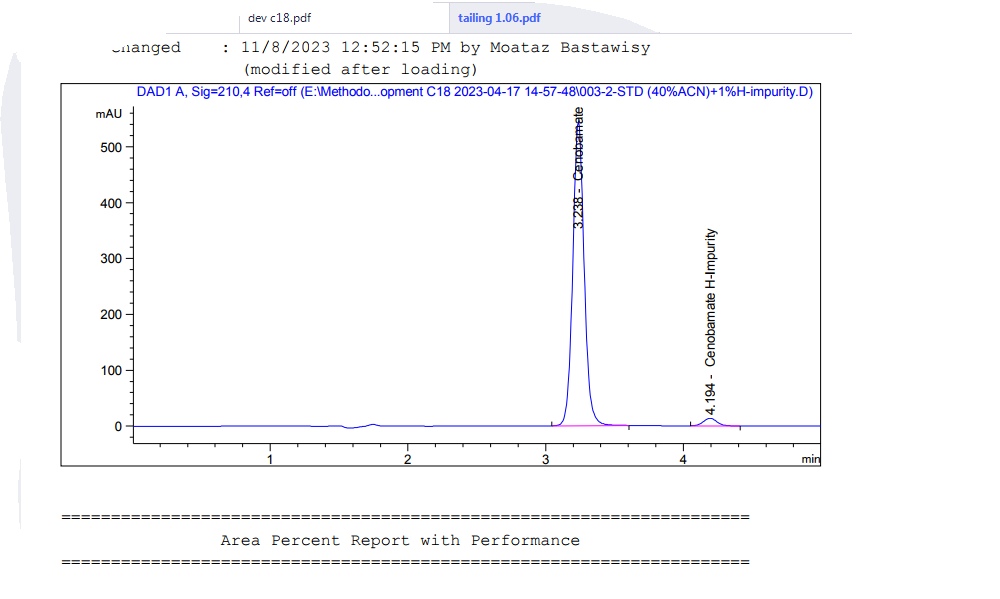


1. Mobile Phase (Buffer pH 3 : methanol) 50:50


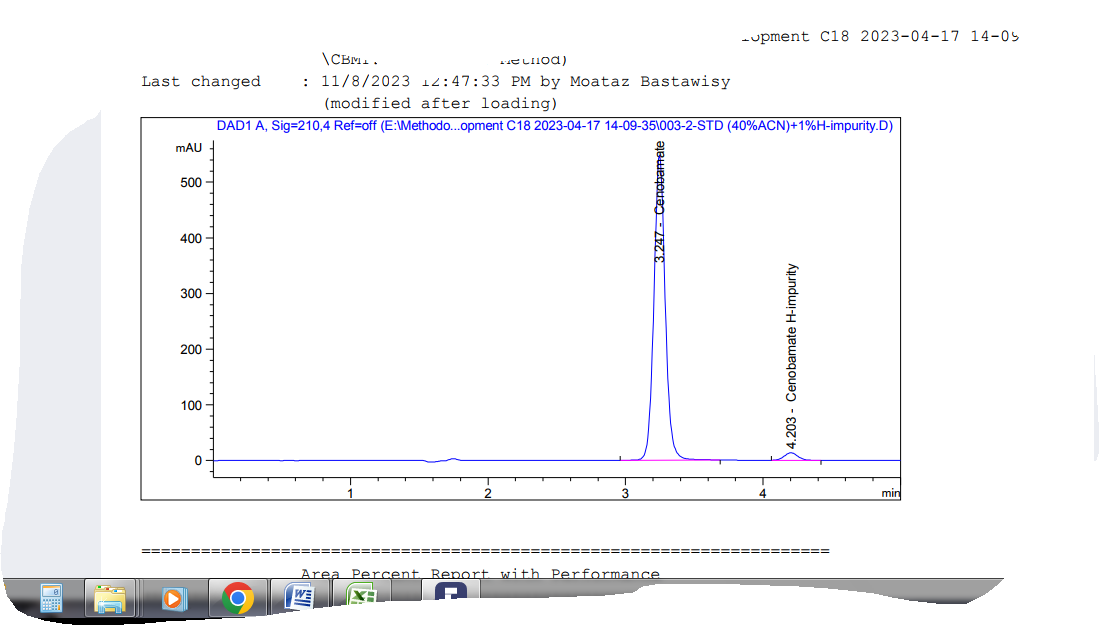


**b)**  Mobile Phase (Buffer pH 6: methanol) 50:50


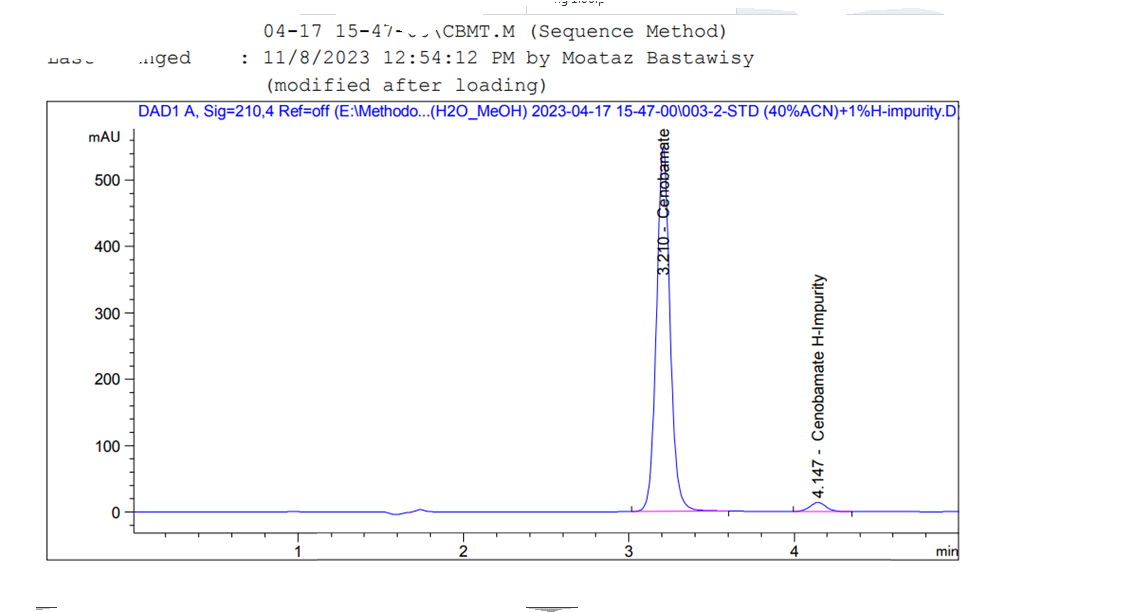


**c)** Mobile Phase (purified water: methanol) 50:50

**Figure S2:** Chromatogram of CNB utilizing phosphate buffer adjusted at pH 3 (a), phosphate buffer adjusted at pH 6 (b), and purified water (c) in the mobile phase.

**
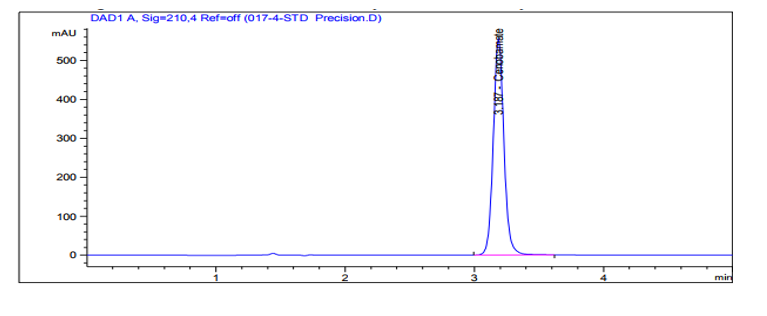
**

**Figure S3: Chromatogram of CNB** **(100 µg.mL^-1^ ) in tablet form at the optimum chromatographic condition.**

**Table S1:** The system suitability parameters for all studied chromatographic conditions for separation of CNB and its H-impurity

| **Capacity factor (*k*)**  **(CNB)** | **Resolution** | **USP Tailing factor** | | **Number of theoretical plates** | | **Retention time** | | **Condition** |
| --- | --- | --- | --- | --- | --- | --- | --- | --- |
|  |  | **H-impurity** | **CNB** | **H-impurity** | **CNB** | **H-impurity** | **CNB** |  |
| **Optimum condition** | | | | | | | | |
| 2.56 | 5.8 | 1.04 | 1.06 | 8369 | 7591 | 4.14 | 3.2 | Thermo, Hypersil BDS C18 column (150x4.6mm, 5µm), mobile phase: Methanol: purified water 50:50, %*v/v* |
| **First trial** | | | | | | | | |
| 4.7 | 3.2 | 1.29 | 1.26 | 8528 | 8003 | 6.35 | 5.51 | Hypersil column BDS C8**,** mobile phase: acetonitrile: phosphate buffer pH 3.0, 30:70, %*v/* |
| **Different stationary phases** | | | | | | | | |
| 2.26 | 4.71 | 1.12 | 1.20 | 8439 | 7698 | 4.0 | 3.26 | **a) Hypersil column BDS C8**, mobile phase: methanol: phosphate buffer pH 3.0, 50:50, %*v/v.* |
| 2.49 | 5.81 | 1.06 | 1.07 | 8553 | 7653 | 4.19 | 3.2 | **b) Hypersil column BDS** **C18,** mobile phase: methanol: phosphate buffer pH 3.0, 50:50, %*v/v.* |
| **Different aqueous composition of mobile phase, using methanol (50%) as organic modifier and Hypersil column BDS C18** | | | | | | | | |
| 2.49  2.52  2.56 | 5.81  5.8  5.8 | 1.06  1.05  1.04 | 1.07  1.06  1.06 | 8553  8595  8369 | 7653  7767  7591 | 4.19  4.20  4.14 | 3.2  3.2  3.2 | **a) Buffer pH 3.0**  **b) Buffer pH 6.0**  **c) Purified water** |

**Table S2:** The percentage of degradation at different degradation conditions

| **Stress condition** | **Degradation %** |
| --- | --- |
| **Acid degradation ( 1mL of 3.0 M HCl at 60 ^0^C)**  2 hrs  3 hrs  4 hrs | 0.00  0.00  0.00 |
| **Acid degradation (1mL of 6.0 M HCl at 80 ^0^C (**  2 hrs  3 hrs  4 hrs  20 hrs* | 0.00  0.00  0.64  2.43 |
| **Base degradation (2mL of 0.005 M NaOH at 40 ^0^C)**  5 min  10 min  15 min  20 min  30 min | 0.30  3.10  3.56  4.79  9.73 |
| **Base degradation (2mL of 0.005 M NaOH at 60 ^0^C)**  5 min*  10 min  15 min  20 min  30 min  **Photo degradation** | 11.24  29.24  33.17  41.60  46.44 |
| **Direct sun light (**6 hrs)  **Direct Lab Light (1070 lux.h) (**6 hrs) | 0.00  0.00 |
| **Oxidative degradation, (2mL of H_2_O_2_ 30% w/w, at room temperature)**  0.2 mL  0.5 mL  1.0 mL  2.0 mL  3.0 mL | 0.00  0.00  0.00  1.00  1.20 |
| **Oxidative degradation, (2mL of H_2_O_2_ 30% w/w, at 60 ^0^C)**  30 min  60 min  2 hrs  3 hrs  4 hrs* | 0.00  1.16  0.39  2.29  5.40 |
| **Heat Degradation**  3 hrs, at 80 ^0^C  6 hrs, at 80 ^0^C* | 0.90  5.90 |

* Optimum stress Conditions

**Table S3** : Comparison between the proposed method and a previously published stability indicating method regarding the oxidative and alkaline degradation

|  | **The published method ^[6]^** | | | **The proposed method** | | |
| --- | --- | --- | --- | --- | --- | --- |
| **Oxidation stress degradation** | **Condition** | **Time** | **% degradation** | **Condition** | **Time** | **% degradation** |
|  | 1 ml of 20% H_2_O_2_ at 60 ̊C | 30 min | 6.26% | 2mL of 30% H_2_O_2_ , at 60 ̊C | 4 hrs | 5.40% |
| **Alkaline stress degradation** | 1 ml of 2N NaOH at 60 ̊C | 30 min | 2.20% | 2mL of 0.005 M NaOH at 60 ̊C | 30 min | 46.44% |

**Table S4:** Results of accuracy studies of CNB assay in pure drug and in spiked placebo

| **Samples** | **Conc. taken**  **(μg. mL^−1^)** | **Mean conc. found***  **(μg. mL^−1^)** | **%**  **Recovery** | **Mean % recovery**  **± SD** |
| --- | --- | --- | --- | --- |
| **Pure form** | 50 | 50.0030 | 100.01 | 99.93 ± 0.097 |
|  | 75 | 74.8625 | 99.82 |  |
|  | 125 | 124.9408 | 99.95 |  |
| **Spiked placebo** | 80 | 79.10 | 98.88 | 99.68 ± 0.703 |
|  | 100 | 99.98 | 99.98 |  |
|  | 120 | 120.23 | 100.19 |  |

*Average of three determination

**Table S5:** Results of precision study for assay of CNB in pure form

| **Repeatability** | | | | | **Intermediate precision** | | | | |
| --- | --- | --- | --- | --- | --- | --- | --- | --- | --- |
| **Conc. taken**  **(µg. mL^-1^)** | **Conc. found**  **(µg. mL^-1^)** | **Mean conc. found***  **(µg. mL^-1^)** | **SD** | **RSD**  **(%)** | **Conc. taken**  **(µg. mL^-1^)** | **Conc. found**  **(µg. mL^-1^)** | **Mean conc. found***  **(µg. mL^-1^)** | **SD** | **RSD**  **(%)** |
| 100.0 | 99.20 | 98.85 | 0.53 | 0.54 | 100.0 | 99.91 | 99.80 | 0.47 | 0.47 |
| 100.0 | 98.10 |  |  |  | 100.0 | 100.51 |  |  |  |
| 100.0 | 99.31 |  |  |  | 100.0 | 99.31 |  |  |  |
| 100.0 | 98.69 |  |  |  | 100.0 | 99.76 |  |  |  |
| 100.0 | 99.40 |  |  |  | 100.0 | 99.28 |  |  |  |
| 100.0 | 98.43 |  |  |  | 100.0 | 100.06 |  |  |  |
|  |  |  |  |  |  |  |  |  |  |

*Average of six determinations

**Table S6:** The results of assay of CNB in dosage form

| **Conc. taken**  **(μg. mL^−1^)** | **conc. found**  **(μg. mL^−1^)** | **%**  **Recovery** | **Mean % recovery**  **± SD** |
| --- | --- | --- | --- |
| 100 | 99.43 | 99.43 | 99.13±0.397 |
| 100 | 98.69 | 98.69 |  |
| 100 | 99.31 | 99.31 |  |

**Table S7:** The results of robustness study of CNB assay

|  | **Conc. taken**  **(µg. mL^-1^)** | | **Tailing factor** | **Theoretical plates (N)** | **Mean**  **% Recovery **** | **SD** | **RSD**  **%** | **Pooled RSD**  **(%)** |
| --- | --- | --- | --- | --- | --- | --- | --- | --- |
|  | | **Flow rate (mL. min^-1^)** | | | | | | |
| **0.95**  **1.00***  **1.05** | 100.0 | | 1.07308  1.08777  1.08026 | 7759.7  7405.0  7127.0 | 99.523  98.564  98.240 | 1.253  1.131  0.99 | 1.259  1.148  1.008 | 1.14 |
|  | | **% Organic modifier in the mobile phase** | | | | | | |
| **45**  **50***  **55** | 100.0 | | 1.07308  1.08961  1.16125 | 7807.00  7432.00  7225.00 | 98.073  98.564  98.135 | 1.167  1.131  1.107 | 1.190  1.148  1.129 | 1.15 |

*Optimum chromatographic conditions

**Average of three determinations

**Table S8:** Stability of CNB stock standard solution

| **Standard stock solution** | **Conc. taken**  **(µg. mL^-1^)** | **Mean Conc. Found***  **(µg. mL^-1^)** | **Mean (%)Recovery*** | **RSD (%)** |
| --- | --- | --- | --- | --- |
| **Freshly prepared** | 100.00 | 100.63 | 100.63 | 0.57 |
| **After 48 hrs of preparation** | 100.00 | 100.34 | 100.34 | 0.11 |

*Average of six determination

**Table S9:** Illustration of GAPI results for the developed HPLC method

| **Sample preparation** | | |
| --- | --- | --- |
| Collection (1) | Off-line | red |
| Preservation (2) | None | green |
| Transport (3) | None | green |
| Storage (4) | None | green |
| Type of method direct or indirect (5) | Simple procedures as filtration | yellow |
| Scale of extraction (6) | Macroextraction | Red |
| Solvents/reagents used (7) | Some non green solvents/reagents used | Red |
| Additional treatments (8) | None | Green |
| **Reagent and solvents** | | |
| Amount (9) | 10-100 mL | Yellow |
| Health hazard (10) | Moderately toxic; could cause temporary  incapacitation; NFPA = 2 or 3 | Yellow |
| Safety hazard (11) | Highest NFPA flammability or instability score of2 or 3, or a special hazard is used | Yellow |
| **Instrumentation** | | |
| Energy (12) | ≤1.5 kWh per sample | Yellow |
| Occupational hazard (13) | ­­­­­----------------- | Yellow |
| Waste (14) | >10 mL | Red |
| Waste treatment(15) | No treatment | Red |
| **Additional mark** | | |
| Circle in the middle of GAPI: procedures for qualification and quantification | | |
